# Massive hydrothorax following subclavian vein catheterization

**DOI:** 10.1186/1755-7682-3-32

**Published:** 2010-11-15

**Authors:** Hesham R Omar, Ahmad fathy, Mohamed Elghonemy, Rania Rashad, Engy Helal, Devanand Mangar, Enrico Camporesi

**Affiliations:** 1 Department of Cardiology, Cairo University Hospital. Cairo, Egypt; 2 Department of Cardiology, National Heart Institute, Cairo, Egypt; 3 Critical Care Department, Cairo University Hospital, Cairo, Egypt; 4 Emergency Department, Elagouza Hospital, Cairo, Egypt; 5 Department of Anesthiology, Tampa General Hospital, Tampa, Florida, USA; 6 Department of Surgery/Anesthesiology, University of South Florida, Tampa, Florida, USA; 7 Department of Molecular Pharmacology and Physiology, University of South Florida, Tampa, Florida, USA

## Abstract

Since the introduction of central venous catheterization for monitoring of the venous pressure, fluid infusion and hyperalimentation, the literature has been full of serious life-threatening complications. Of these complications is the false positioning of the central venous catheter and subsequent development of pleural effusion. In this report we are describing a case of iatrogenic massive pleural effusion following subclavian vein catheterization necessitating intercostal tube drainage and mechanical ventilation. The case highlights the importance of ensuring adequate positioning of the catheter after insertion through aspiration of venous blood, immediate post insertion X-ray and the utilization of ultrasound guidance in cases with expected difficult catheterization.

## Introduction

Massive pleural effusion due to intrapleural pouring of fluids administered to the patient has been rarely reported. We are describing a case of massive hydrothorax that developed after the insertion of a subclavian catheter and administering fluids through the line requiring immediate intercostal tube insertion and mechanical ventillation. The fluid was a clear fluid that could be aspirated from the catheter as well confirming the diagnosis.

## Case Report

**Figure 1:**
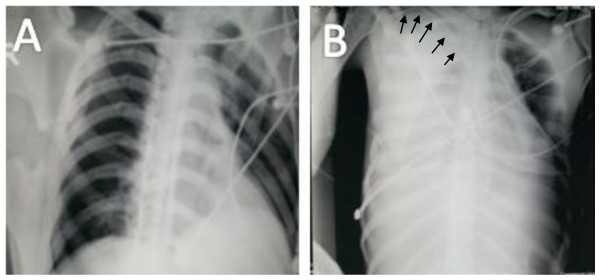
**Revealing a normal anteroposterior chest radiograph before subclavian catheterization (panel A)**. chest X-ray performed after catheterization revealing massive left sided pleural effusion with mediastinal shift to the left side. Arrows point to the course of the catheter.

A 23 year old male was transferred to the ICU one month after a fall from height and head injury. On admission the patient was in a persistant vegetative state with clinical evidence of dehydration. The patient was on 5 litres O_2 _per minute via trachestomy and arterial blood gases were within normal limits. Clinical examination revealed evidence of dehydration. Chest X-ray showed no abnormalities as shown in figure 1a.

To correct dehydration and monitor volume status, a central venous line was inserted. Right subclavian approach was used. Four attempts to cannulate the subclavian vein were performed by a skilled intensivist using an 18 gauge needle but were all unsuccessful. During these attempts there was neither arterial puncture nor air aspiration. After getting little backflow of blood which was dark and nonpulsatile, ensuring a venous position of the needle, An 0.035" guidewire was then introduced afterwhich a dilator was advanced for 5 cm without apparent resitance. This was followed by sliding of a 7 french catheter over the guidewire, and the wire was then withdrawn without any resistance. Despite the smooth insertion of the catheter, there was a negative aspiration of blood which we attributed to severe dehydration. Central venous pressure was measured after insertion and was -1 mmHg so one liter of colloid was infused followed by 1 liter of crystalloids over a period of 12 hours.

Several hours later, the patient started experiencing severe respiratory distress with rise in the central venous pressure from -1 mmHg to 16mmHg. Clinical examination revealed a respiratory rate of 40/minute and absent air entry on the right hemithorax as well as dullness to percussion. An arterial blood gas revealed hypoxia and combined metabolic and respiratory acidosis with Po2 of 49 mmHg, oxygen saturation 79%, pco2 54.9, PH 7.11 and HCo3 17.2. The patient started to develop severe bradycardia and hypotension necessitating atropine and mechanical ventilation. Bedside anteroposterior chest radiograph was performed as shown in figure 1 panel B, which revealed the presence of massive right side pleural effusion and mediastinal shift to the left. The presence of pleural effusion was confirmed by syringe aspiration of clear fluid. A 28- French intercostal tube connected to underwater seal was then inserted followed by gradual withdrawal of the pleural fluid which was continuously clear till 3 liters were withdrawn over several hours. This was the same amount of fluid given to the patient through the subclavian catheter for correction of dehydration. At that time aspiration from the central venous catheter revealed the same clear fluid as that was drawn from the intercostal tube raising the suspicion of the intrapleural position of the venous catheter. The catheter was then removed from the subclavian vein and reinserted in the left internal jugular vein through an anterior approach. After withdrawal of the pleural fluid, marked relief of the respiratory distress and improvement of the oxygenation ensued.

**Figure 2:**
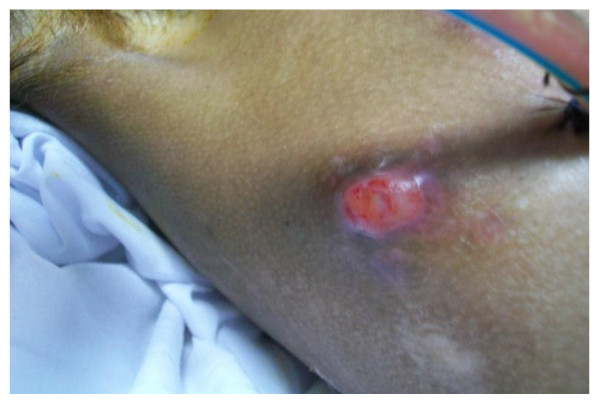
**Demonstrating the site of the pleurocutaneous fistula**.

3 weeks later the patient was readmitted to the ICU for increasing difficulty in breathing. On examination, the patient was tachypnic with diminished air entry over the right lung and dullness to percussion and a purulent fluid coming from an opening in his right chest wall as shown in figure 2. Diagnostic aspiration of the pleural fluid revealed frank pus. Analysis of the pleural fluid revealed low glucose and PH and culture revealed methicillin resistant staphylococcus aureus. An intercostals tube was placed for drainage and the patient was started on vancomycin therapy.

## Discussion

Central venous lines represent an imperative tool in the ICU for delivering fluids, nutrients, drugs and monitoring the volume status. Hazards of central venous catheterization involve immediate and remote complications. The immediate complications are usually mechanical in the form of pneumothorax, hemothorax [1] or arterial puncture. Remote complications are mostly due to infection of the central line. However delayed presentations can occur in the form of pericardial effusions, cardiac tamponade [2] and pleural effusions. In this report we are describing the occurrence of an iatrogenic hydrothorax following subclavian vein catheterization which was later complicated by empyema a pleurocutaneous fistula. These delayed complications are not less serious and any delay in diagnosis can be catastrophic. Careful insertion techniques, as well as continued vigilance regarding the correct position and function of central venous catheters, are imperative to help prevent serious complications.

On the contrary to internal jugular cannulation, subclavian vein is sometimes more accepted or tolerated by patients and is relatively a safe procedure when performed by an experienced physician. This is because of the large caliber with an estimated diameter of 2 cm and running a fixed course. The major concern though is the risk of pneumothorax because at some points, the subclavian vein is only 5 mm above the apical pleura of the lung. In our case, we've experienced a rare complication of subclavian vein catheterization due to massive pleural effusion.

In our patient the clear colour of the fluid retrieved from the intercostals tube, the aspiration from the subclavian line of a fluid identical to that retrieved from the intercostal tube (especially after a negative aspirate from the subclavian catheter in the beginning) and the stoppage of effusion after removal of the central line all confirmed the intrapleural pouring of fluids administered through the catheter. This complication has been previously described by Ciment et.al [3]. however the effusion in that case was contralateral. Contralateral effusions are due to mediastinal leakage and not direct intrapleural insertion.

In our patient the clear colour of the fluid retrieved from the intercostals tube, the aspiration from the subclavian line of a fluid identical to that retrieved from the intercostal tube (especially after a negative aspirate from the subclavian catheter in the beginning) and the stoppage of effusion after removal of the central line all confirmed the intrapleural pouring of fluids administered through the catheter. This complication has been previously described by Ciment et.al [3]. however the effusion in that case was contralateral. Contralateral effusions are due to mediastinal leakage and not direct intrapleural insertion.

The key to expecting complications is the presence of a negative aspiration which is usually a marker of a false pathway. There are several causes that can explain a negative aspiration: First, external or internal kinking of the catheter, second, compression by the muscles and third is the passage of the catheter through a false lumen which we assume was the situation in our case. Urgent chest X-ray is therefore mandatory in any case with negative aspiration of blood after central venous catheter insertion. Management involves drainage of the pleural fluid, maintaining the patient's hemodynamics, and adequate oxygenation in addition to removal of the improperly placed catheter.

This report aims to sensitize the readers to this complication and create awareness among intensivists and anasthesiologists to the importance of ensuring a correct position of the catheter by aspiration of venous blood and immediate post insertion X-ray confirming a correct position and the use of ultrasonographic guidance whenever available especially in cases with expected difficult catheterization.

## Competing interests

The authors declare that they have no competing interests.

## Authors' contributions

HO and AF are responsible for drafting the manuscript and literature search. DM and EC, EH and ME have made critical revisions to the manuscript. All authors have read and approved the final manuscript.

## Consent

Written informed consent was obtained from the patient's next of kin for publication of this case report. A copy of the written consent is available for review by the Editor-in-Chief of this journal
